# Proton Therapy for Intracranial Meningioma for the Treatment of Primary/Recurrent Disease Including Re-Irradiation

**DOI:** 10.3389/fonc.2020.558845

**Published:** 2020-12-14

**Authors:** Damien C. Weber, Nicola Bizzocchi, Alessandra Bolsi, Michael D. Jenkinson

**Affiliations:** ^1^ Center for Proton Therapy, Paul Scherrer Institute, ETH Domain, Villigen, Switzerland; ^2^ Radiation Oncology Department, University Hospital Zürich, Zürich, Switzerland; ^3^ Radiation Oncology Department, University Hospital of Bern, Inselspital, Bern, Switzerland; ^4^ Department of Neurosurgery, The Walton Centre NHS Foundation Trust, Liverpool, United Kingdom; ^5^ Institute of Translational Medicine, University of Liverpool, Liverpool, United Kingdom

**Keywords:** meningioma, proton therapy, recurrent disease, primary treatment, reirradiation, pencil beam scanned proton therapy, surgery

## Abstract

Meningeal tumors represent approximately 10–25% of primary brain tumors and occur usually in elderly female patients. Most meningiomas are benign (80–85%) and for symptomatic and/or large tumors, surgery, with or without radiation therapy (RT), has been long established as an effective means of local tumor control. RT can be delivered to inoperable lesions or to those with non-benign histology and for Simpson I–III and IV–V resection. RT can be delivered with photons or particles (protons or carbon ions) in stereotactic or non-stereotactic conditions. Particle therapy delivered for these tumors uses the physical properties of charged carbon ions or protons to spare normal brain tissue (i.e. Bragg peak), with or without or a dose-escalation paradigm for non-benign lesions. PT can substantially decrease the dose delivered to the non-target brain tissues, including but not limited to the hippocampi, optic apparatus or cochlea. Only a limited number of meningioma patients have been treated with PT in the adjuvant or recurrent setting, as well as for inoperable lesions with pencil beam scanning and with protons only. Approximately 500 patients with image-defined or WHO grade I meningioma have been treated with protons. The reported outcome, usually 5-year local tumor control, ranges from 85 to 99% (median, 96%). For WHO grade II or III patients, the outcome of only 97 patients has been published, reporting a median tumor local control rate of 52% (range, 38–71.1). Only 24 recurring patients treated previously with photon radiotherapy and re-treated with PT were reported. The clinical outcome of these challenging patients seems interesting, provided that they presented initially with benign tumors, are not in the elderly category and have been treated previously with conventional radiation dose of photons. Overall, the number of meningioma patients treated or-re-irradiated with this treatment modality is small and the clinical evidence level is somewhat low (i.e. 3b–5). In this review, we detail the results of upfront PT delivered to patients with meningioma in the adjuvant setting and for inoperable tumors. The outcome of meningioma patients treated with this radiation modality for recurrent tumors, with or without previous RT, will also be reviewed.

## Introduction

Meningiomas are the commonest primary brain tumor and account for 10–25% of all cases (1). The WHO classification describes three different histological grades: WHO grade I meningioma account for up to 80% of cases and have a low recurrence rate; WHO grade II comprise approximately 20–30% of cases and have a recurrence rate of ~30–40%; and grade III meningioma comprise around 1–2% and invariably recur (2). Asymptomatic and incidental meningiomas do not usually require active treatment and can be safely monitored (3, 4). However, for symptomatic or growing tumors, surgery is still the primary treatment modality, can achieve long-term tumor control and in some cases can be curative (5). However, despite advances in surgical techniques not all meningioma are appropriate for surgery (e.g. due to anatomical location) nor are all meningioma amenable to complete resection (e.g. due to proximity to critical neurovascular structures or tumor consistency). Furthermore, even when meningiomas are completely resected, recurrence can still occur. In meningiomas that recur, surgery is more challenging due to scar tissue, and is associated with higher rates of morbidity and mortality. Recently several integrated molecular models to predict the risk of recurrence risk have been developed (6, 7), which, once prospectively validated prospectively, could be used to guide to the use of adjuvant radiotherapy. Due to the risk of recurrence, radiotherapy has a clear role in the management of meningioma in order to achieve durable, long-term control. It is used in the adjuvant setting for most malignant meningioma, for some atypical meningioma and for the occasional benign meningioma. Currently the standard modality is fractionated external beam radiotherapy with photons or radiosurgery for small tumors that are not in direct vicinity of critical structures. The expansion of proton beam facilities has led to increased use of this modality. The main difference between photons, delivered in stereotactic- or non-stereotactic condition, and protons is the remarkable dose distribution obtained with the latter, where the dose is delivered at a narrow area at the distal end of the proton trajectory (i.e. the Bragg Peak). For small target volumes, it may be questionable if proton therapy (PT) delivered with a Gantry obtains a better dose distribution than radiosurgery (8), for larger tumors, for which this latter treatment modality is not an option, protons usually always achieve an improved dose-conformation when compared to photons. The aim of this review is to describe the contemporary experience of using PT for the treatment of intracranial benign- and non-benign meningioma.

## Proton Therapy for Brain Tumors

**Figure 1:**
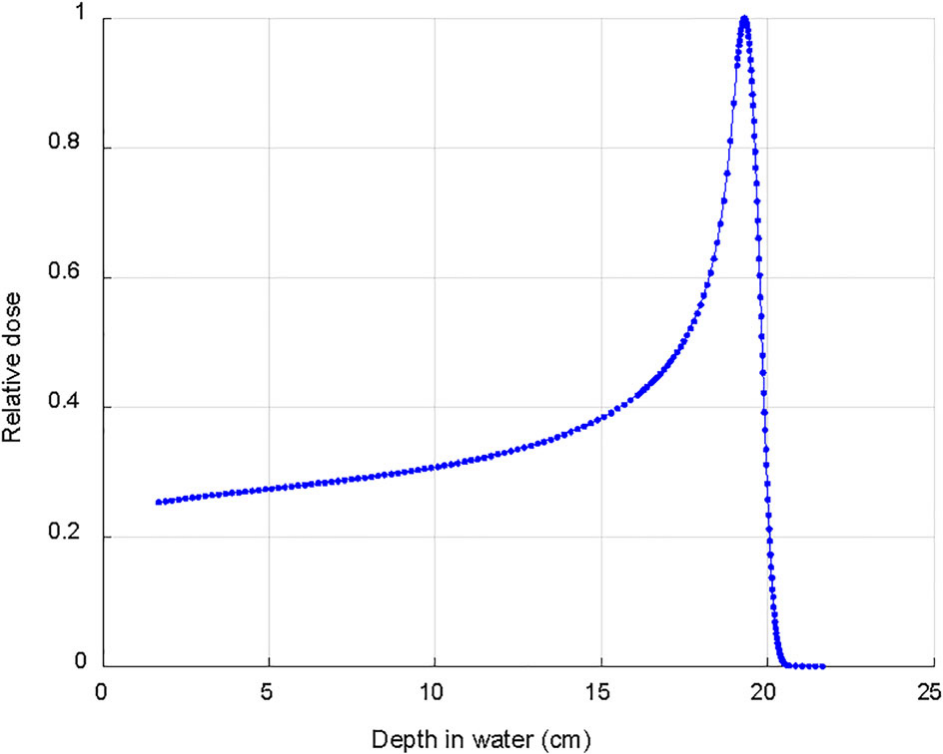
Typical depth dose curve in water for a clinical 170 MeV proton beam used to treat intracranial meningioma.

**Figure 2:**
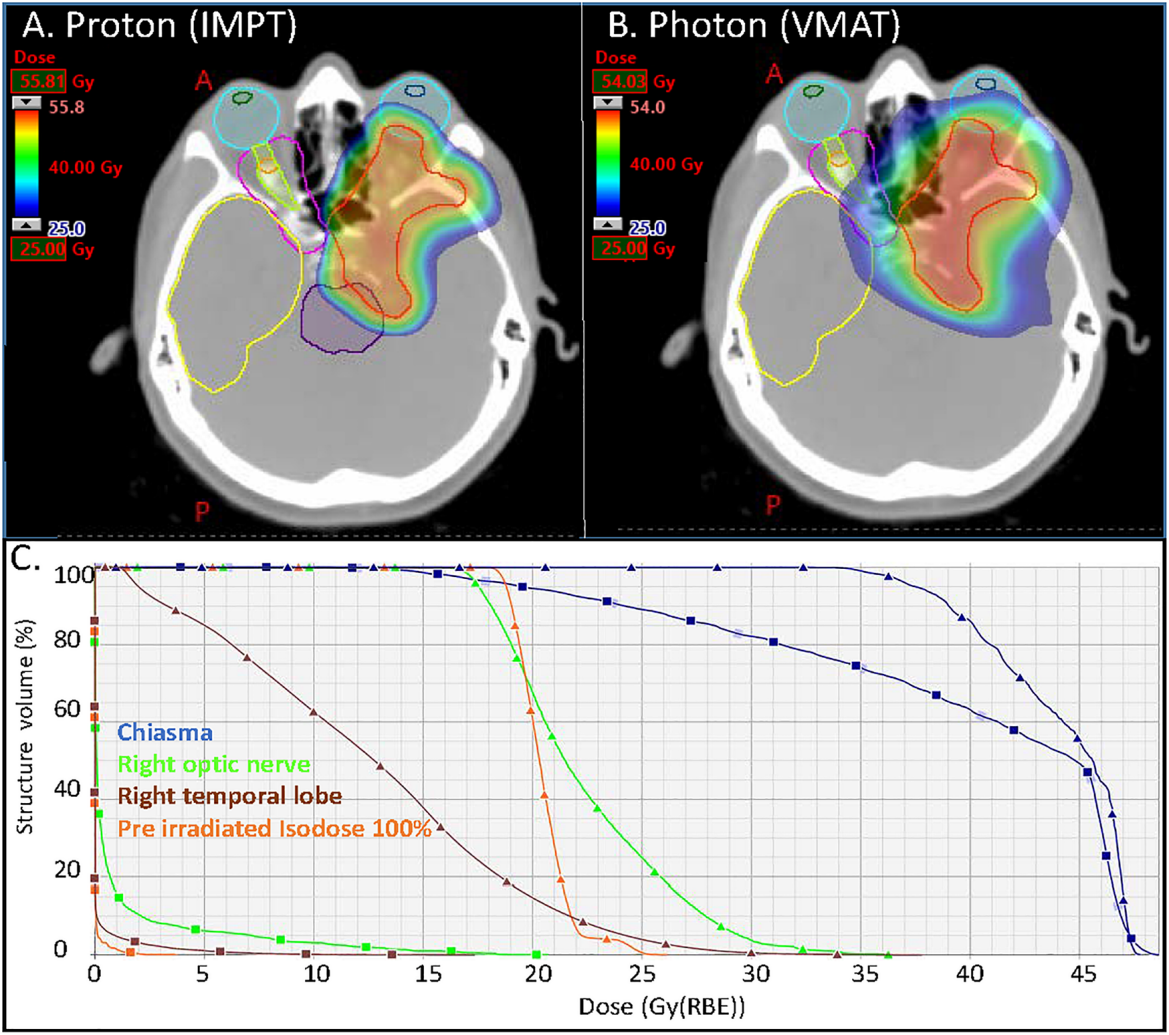
Axial distributions for the **(A)** proton (IMPT) and **(B)** photon (VMAT) plans. In magenta and orange the isodoses lines of the pre irradiation (50% and 100% of 54 Gy respectively); **(C)** DVHs of the optic nerve right, chiasma, right temporal lobe and ROI defined within the 100% isodose line of the pre irradiation (square markers for the proton plan and triangular markers for the photon plan). VMAT, Volumetric modulated Arc Therapy (photon plan); IMPT, Intensity Modulated Proton Therapy (Proton plan).

The dose deposition in tissue of proton beams is described by a sharply defined Bragg peak, where the bulk of the dose is deposited; beyond the peak the deposited dose drops to zero within a few millimeters (9) (**Figure 1**). The maximum depth (proton range) depends only on the initial energy of the proton beams. The resulting PT dose distributions present both superior dose conformality and lower total integral dose when compared to the photon ones. Intensity Modulated Proton Therapy (IMPT) technique (10), available for Pencil Beam Scanning (PBS) systems, can achieve particularly steep dose gradients, thus increasing furthermore the PT advantages. Brain tumors, including meningiomas, typically located in close proximity to many critical organs at risk (OAR), can clearly benefit from PT dose conformality and healthy tissue sparing (11). This rationale is further supported by the dose-dependent relationship for many radiation induced toxicities which develop after RT for brain tumors. This is the case, for instance, of dose to the hippocampus which correlates with memory outcomes (12) and dose to the hypothalamus and pituitary which correlates with the severity of endocrine dysfunction (13). The increased OAR dose sparing and integral dose reduction typical of PT is even more crucial in cases of re-irradiation where PT is frequently the only possible treatment modality. **Figure 2** details such a case treated at the Paul Scherrer Institute with 52.2 GyRBE administered after a photon irradiation for tumor recurrence. Of note, the second irradiation with protons could completely spare the contralateral temporal lobe and optic nerve (**Figure 2**). As shown in the dose–volume histogram (**Figure 2**), PT enabled complete sparing of the initial target volume (i.e. pre-irradiated Isodose line 100%) treated with photons and thus made re-irradiation possible. Many factors such as the tumor location (14), size and shape of the target volumes influence the magnitude of the PT dosimetric advantages compared to photons. A very recent study (15) for skull base meningiomas, comparing VMAT, IMRT and IMPT reported a significant mean dose reduction up to 48% for the bilateral hippocampi for IMPT as compared to VMAT. Similar differences were found when comparing mean dose to the normal brain tissue; the comparison between IMRT and IMPT resulted in even larger differences in dose to OAR, thus possibly leading to a clinically relevant reduction of late neurocognitive side effects.

PT can be administered to meningioma patients with several delivery paradigms. Historically, protons were delivered with a passive scattering system with which the distal end of the proton tracks are controlled/modulated through the use of compensators and the lateral aspect of the proton beam is shaped by brass apertures. In contrast, spot-scanning PT, or as it is called currently pencil beam scanning (PBS) PT, pristine proton pencil beams are scanned in the target volume with different energies to achieve the wanted depth dose distributions. This is in essence a discrete way of administering dose using a ‘step and shoot’ paradigm. A US group has shown that PBS achieved a better cochlear and lens sparing when compared to passive scattering delivery in brain tumor patients treated with cranio-spinal irradiation (16). Using the same delivery model, radiation volume is virtually divided into numerous slices in dynamic raster scanning, which are subdivided into voxel points. These slices are scanned sequentially but continuously using the focused particle pencil beam.

The steep dose gradients of IMPT, essential to achieve very high dose conformality, are sensitive to range and setup uncertainties, hence potentially affecting the quality of PT delivered dose distributions. Those effects can be mitigated by various techniques, the most effective ones being robust planning (17) and robust optimization (18). The radiation biological characteristic of protons is also a concern in PT, where a constant generic relative biological effectiveness (RBE) value of 1.1 is clinically used. It is anyway well known that RBE increases with increasing Linear Energy Transfer (LET), thus presenting with the highest value in the distal fall-off (19). As proton RBE values are still associated with considerable uncertainties, radiobiological evaluation of PT plans focus preferably on linear energy transfer (LET), a physical parameter which can be calculated quite accurately based on treatment planning information. Adjustment of treatment fields’ direction or LET optimization of PT plans can contribute to LET reduction in case of high LET areas localized in critical structures. Research developments in these areas of PT are likely going to further increase its clinical benefits.

## Proton Therapy for WHO Grade I Meningioma

**Table 1** details the PT series delivered to WHO grade I meningioma. Of note, approximately half (*n* = 237; 45.5%; **Table 1**) of the meningioma patients were treated with either hypo-fractionated stereotactic PT (SFPT) or with proton radiosurgery (pSRS). For non-SFPT/pSRS series, the Paul Scherrer Institute recently published the results of 61 WHO grade I meningioma patients treated with pencil beam scanning PT to a median dose of 54 GyRBE (**Table 1**) (22). For those patients progressing/recurring, most of them failed within the treatment field. The estimated tumor local control (LC) and overall survival (OS) was 95.7 and 92.1%, respectively (**Table 1**). The difference in LC rates between benign and non-benign tumors were significantly different (*p* <0.01) in this PT cohort. Only 1/10 WHO grade I patients had a grade CTCAE 3 adverse event during follow-up. Wenkel et al. reported on 46 WHO grade I meningioma patients (median age, 50 years; range, 11–74) treated with combined photon-proton radiotherapy (26). The ratio of median photon and proton dose was 18.4%, but some patients were treated with photon > proton doses depending on the availability of the proton treatment unit on the Harvard–Cambridge campus. Most tumors (29/46; 63%) were treated for recurrence, either after subtotal (*n* = 19) or gross total resection (*n* = 10). Only nine (20%) patients were treated postoperatively with protons. Of note, the dose level delivered by the Boston group is substantially higher (median, 59 GyRBE; **Table 1**) than other groups treating these patients with protons worldwide. After a median follow-up of 53 months (range, 12–207) the estimated recurrence-free- and OS were 88 and 77%, respectively. The 10-year toxicity-free survival was 80%. A substantial number of patients (4/46; 8.7%) presented with visual/ocular toxicity (**Table 1**), and dosimetric analysis revealed that these patients received a maximum median dose of 63.2, 67.5 and 67.4 GyRBE to the Chiasma, Optic nerve left- and right, respectively. Of note, no patient died of progressive disease but one patient died (CTCAE grade 5) of brain necrosis 22 months after therapy. Slater et al. reported another series on 72 skull-base WHO grade I-II meningioma and the outcome of 47 patients with benign tumors (25) was detailed in this paper. The median total doses in the entire cohort for patients (age range, 9–87 years) with and without histologic verification were 59 and 57 GyRBE (range, 50.4–66.6). With a median follow-up of 74 months (range, 3–83), the estimated 5-year LC was 99%. Overall, 6 patients developed radiation induced toxicity, which included visual adverse events (n = 3) and brain necrosis (n = 2). The Heidelberg group reported on 102 skull-base histologically proven (WHO grade I, *n* = 60) or image-defined meningioma patients treated with proton therapy using the raster scanning delivery paradigm (28). The median age of patients (80% female) was 52 years (range 45–59) and after a median follow-up of 46.8 months, four local progressions were observed. As a result of the small number of events, the median PFS was not reached. The estimated 5-year PFS and OS were 96.6 and 96.2%, respectively (**Table 1**). Three (2.7%) patients developed brain necrosis, of which two were symptomatic, but no visual toxicity was observed. Finally, another skull-base meningioma series was published by the Orsay group in Paris reporting on the outcome of 51 patients (42 females; 82.4%) (23). Forty-four (86.3%) patients had histologically proven WHO grade I meningioma and the median age of patients was 56 years (range, 11–75). The mean/median follow-up period was 25.4/21.0 months with a range of 1–90 months and only local failure was observed. The estimated 4-year LC and OS were 98 and 100%, respectively.

**Table 1:** Pencil beam scanning or passive-scattering proton therapy delivered to presumed or histologically-proven WHO grade I tumors.

Author	#Ref	Year	#pts	Median tumor^∞^/target volume^⌂^(cm^3^)[range]	Mean/median follow-up period (months)	Dose (GyRBE)(median/mean)	Delivery modality	Tumor outcome	Proton only	Visual toxicity^#^(%)	Brain necrosis^ŧ^(%)
^⌂⌂^Vlachogiannis et al.	(20)	2017	170	^⌂^13.0 [1–64]	84.0	14–46 ^¶^ [21.9]	PSPT	PFS^***^: 85%	Yes	5/170 (2.9%)	5/170 (2.9%)
El Shafie et al.	(21)	2018	102	NR	46.8	50–60 [50.4]	Raster scanning	PFS^**^: 96.6%	Yes	0/102 (0%)	3/102 (2.7%)
Murray et al.	(22)	2017	61	^∞^ 21.4[0–547]^○^	56.9	50.4–56.0 [54.0]	PBS only	LC^**^: 95.7%	Yes	7/96^¥^ (7.3%)	3/96^¥^ (3.1%)
Noel et al.	(23)	2005	51	NR	25.4	54–64 [60.6]	PSPT only	LC^**^: 98%	No	0/51 (0%)	0/51 (0%)
^⌂⌂^Halasz et al.	(24)	2011	50	^∞^ 2.1[0.3–9.7]	32	10.0–15.5^¶^ [13.0]	PBS only	LC^*^: 94%	Yes	0/50 (0%)	2/50 (4%)
Slater et al.	(25)	2012	47	^⌂^27.6[1–224]	74.0	50.4–66.6	PSPT only	LC^**^: 99%	Yes	3/72^¥^ (4.2%)	2/72^¥^ (2.8%)
Wenkel et al.	(26)	2000	46	^∞^ 32[2–243]	53.0	53.1–74.1 [59.0]	PSPT only	RFS^***^: 88%	No	4/46 (8,7%)	4/46 (8.7%)
^⌂⌂^Vernimmen et al.	(27)	2001	23	^⌂^15.6[2.6–63]	40.0	54–61.6 17.3–24.3^¶^ [20.3] ^¶^	PBS only	LC^**^: 88%^¶^	Yes	0/27 (0%)	1/27 (3.7%)
Total # patients			**521**								
**Median % LC/PFS** (Range)								**96%** (85–99%)			
**Median % Toxicity** (Range)										**2.6%** (0.0–8.7%)	**3.4%** (0.0–8.7%)

Pts, patients; PBS, Pencil Beam Scanning proton therapy; PSPT, Passive-Scattering Proton Therapy; NR, not reported; LC, Local Control; RFS, Recurrence-free Survival; PFS, Progression-free survival.

^*^3-year.

^**^4/5-year.

^***^10 year.

^¥^Total number of WHO grades I–III patients in cohort.

^¶^Stereotactic condition/radiosurgery/Hypo-fractionated PT, horizontal beam.

^#^Including but not limited to ocular adverse events, retinopathy and optic neuropathy.

^ŧ^Radiological brain edema/biopsy proven brain necrosis/epilepsy.

^∞^Tumor volume.

^⌂^Target volume/Clinical target volume.

^○^WHO grades I and II.

^⌂⌂^Fractionated stereotactic proton therapy/proton radiosurgery.

Bold values provided are highlighting the overall results of patients numbers and outcome (Toxicity and tumor outcome).

For the SFPT/pSRS series, the largest series originates from the Uppsala group which reported the outcome of 170 WHO grade I meningioma patients (mean age, 54.2 years; 22–85) treated with hypo-fractionated (3–4 GyRBE per fraction) SFPT delivered with a horizontal beam (20). Most of these benign meningiomas (155/170; 86.1%) were skull base tumors in female (135/170; 79.4%) patients treated with five fractions of 4 GyRBE, due to the limited availability of the Gustav Werner cyclotron of 10 weeks annually. Median delivered dose was 21.9 GyRBE (14–46). After a median follow-up of 84 months, the estimated 5- and 10-year progression-free survival rates were 93 and 85%, respectively. Only 3 (1.7%) patients died of meningioma. Radiation-induced adverse events were seen in 16 (9.4%) patients, with pituitary insufficiency (37.5% of all toxicities) being the most common. Brain radiation necrosis was observed in 5 (2.9%) patients, most (4/5) being asymptomatic (**Table 1**). Older patients and patients with tumors located in the middle cranial fossa had a lower risk for tumor progression. The Boston group has also been delivering pSRS for benign meningioma and reported the outcome of these patients with a median follow-up of 32 months (range, 6–133) (24). One fraction of 10.0–15.5 GyRBE (median, 13.0) was delivered to histologically proven or image-defined <4 cm (median volume, 2.1 cm^3^) meningiomas. Of note, atypical features were observed in 6 (12%) patients but these features did not meet the criteria for a diagnosis of atypical meningioma. Patients with tumor <2 mm from the optic apparatus were not eligible for pSRS. In this series from 2011, the estimated 3-year local tumor control was 94%. Three (6%) patients presented with radiation-induced complications, of which two were brain complications (**Table 1**). Finally, the South African group have reported the results of 23 WHO grade I skull-base meningioma patients (27). SFPT was delivered either in three fractions with mean dose, 20.3 GyRBE to 18 patients. Noteworthy, 16 fractions or more, with a dose range of 54.0–61.6 GyRBE, was delivered to another five patients. Median volume of these meningiomas was 15.6 cm^3^. The median follow-up time was 40 months (range, 13–69). For the SFPT group, two local failures were observed and the estimated 5-year local control was 88%. No events were observed in the fractionated group. One (3.7%) patient in the hypo-fractionated group developed temporal lobe epilepsy.

These data show that protons can either be delivered conventionally, with or without a pencil beam scanning paradigm, or in stereotactic conditions (i.e. SFPT and pSRS). The meningiomas treated with SFPT or pSRS were usually smaller than their non-stereotactic PT counterparts, with a median tumor volume reported in the three series listed in **Table 3** of 13.0, 2.1 and 15.6 cm^3^, respectively. Interestingly, no increased toxicity (visual toxicity or brain necrosis) was observed with SFPT/pSRS when compared to PT (**Table 1**). Additionally, the outcome was also identical, with the lowest PFS/RFS at 10 years of 85 and 88% for SFPT/pSRS and PT, respectively (**Table 1**).

## Proton Therapy for WHO Grade II–III Meningioma

The French group has reported on 24 patients (50% females) with non-benign meningioma (atypical, *n* = 19; malignant, *n* = 5) only treated with postoperative proton/photon therapy (29). Most patients (n = 18; 75%) were treated after subtotal resection to a mean/median total photon/proton dose of 65/68 GyRBE. After a median follow-up time of 32.2 months (range, 1–72), 10 (41.7%) progression/recurrences were observed. The mean RFS interval and estimated 5-year local tumor control were 27.2 months and 46.5%, respectively. Noteworthy, survival was significantly associated with dose, with a cut-off of 60 GyRBE. The relative risk of dying of meningioma was 8.3 (1.2–57; *p* = 0.029) for those not treated at this dose level. One patient developed radiation-induced necrosis 16 months after the delivery of 68 GyRBE-Gy, of which 34 Gy was with photons (**Table 2**). The Paul Scherrer Institute has also reported the outcome of 33 grade II and 2 III meningioma patients treated with protons only (22). The median administered dose was 62 GyRBE with a range of 54 to 68. The majority (9/14; 69%) of all treatment failures from the meningioma cohort were non-benign tumors. The estimated 5-year LC was these tumors was 68%. All but 2 (8/10; 80%) in-field treatment failures were of WHO grade II–III histology. Interestingly, only one brain necrosis (CTCAE grade 3) occurred in a WHO grade II patients (**Table 2**), the other brain necrosis (CTCAE grade 5) and brain edema occurred in WHO grade I patients (**Table 1**). Out of the seven observed visual toxicity, only one occurred in a WHO grade II meningioma patient (**Table 2**). In the Heidelberg series (28), all but two WHO grade II or III meningiomas were treated with carbon ion therapy. The outcome of these two patients treated with protons has not been reported separately. Hug et al. reported on 16 patients treated with photon/proton therapy (**Table 2**). These patients were included in the analysis of a larger (n = 31) cohort of patients with non-benign histology treated with high-dose photon and combined photon/proton therapy (31). Interestingly, the local control of patients treated with protons was significantly (*p* = 0.003) higher than those treated with photons. The same statistical result (*p* = 0.025) applied also for those treated at a dose of 60 GyRBE or higher, regardless of the delivery of photons or protons/photons. In this series, two patients developed symptomatic brain necrosis, one of whom was treated with protons/photons to a dose of 72 Gy RBE (**Table 2**). One last patient treated with 68.4 GyRBE combined photon-proton therapy extensive visual field deficits and retained no functional vision. Finally, Mac Donald et al. reported on 22 WHO grade II meningioma patients (30) treated with protons only. Noteworthy, 6 patients had presumed radiation-induced meningiomas. After a follow-up period of 7–104 months (mean, 39.2), five patients progressed/recurred. The estimated 5-year LC was 71.1% (**Table 2**). The authors have seen the same impact on radiation dose and patient’s outcome as did the French (29) and other US (31) groups. The 5-year LC of those patients treated with > and ≤60 GyRBE were 87.5 and 50%, respectively (*p* = 0.038). One symptomatic CTCAE grade III temporal lobe necrosis was observed (**Table 2**). No SFPT or pSRS delivery was reported for non-benign meningiomas.

**Table 2:** Pencil beam scanning or passive-scattering photon/proton therapy delivered to presumed or histologically-proven WHO grade II-III tumors.

Author	#Ref	Year	#pts	Median tumor^∞^/target volume^⌂^(cm^3^)[range]	WHOgrade	Mean/median follow-up period (months)	Dose (GyRBE)[median/mean]	Delivery modality	Tumor outcome	Protononly	Visual toxicity^#^(%)	Brainnecrosis^ŧ^(%)
Murray et al.	(22)	2017	35	^∞^ 21.4[0–547] ^○^	II–III	56.9^¶^	54–68 [62.0]	PBS	LC**: 68.0%	Yes	1/35 (1.5%)	1/35 (2.9%)
Boskos et al.	(29)	2009	24	^∞^ 48.3[0–120]	II–III	32.2	0–34^¥^ 28.8–68^¥¥^ [68.0]	PSPT	LC**: 46.7%	No	0/24 (0%)	1/24 (4.2%)
McDonald et al.	(30)	2015	22	^∞^ 8.1[0–89.3]	II only	39.0	54–68.4 [63.0]	PSPT	LC**: 71.1%	Yes	0/22 (0%)	1/22 (4.5%)
Hug et al.	(31)	2000	16	NR	II–III	59.0^Ø^	40–72 [62–58] ^#^	PSPT	LC**: 38–52%^#^	No	1/16 (6.3%)	1/16 (6.3%)
Total # patients			**97**									
**Median % LC/PFS** (Range)									**52%** (38.0–71.1)			
**Median % Toxicity** (Range)											**0.8%** (0–6.4)	**4.4%** (2.9–6.3)

Pts, patients; WHO, World Health Organization; PBS, Pencil Beam Scanning proton therapy; PSPT, Passive-Scattering Proton Therapy; NR, not reported; LC, Local Control; RFS, Recurrence-free Survival; PFS, Progression-free survival.

^**^4/5-year.

^¥^Photon dose.

^¥¥^Proton dose.

^¶^Entire meningioma WHO grades I–III cohort.

^#^Mean dose/local control rate for WHO grades II and III, respectively.

^ŧ^Radiological brain edema/biopsy proven brain necrosis/epilepsy.

^Ø^Mean follow-up interval for the photon only and photon/proton treatments.

^∞^Tumor volume.

^⌂^Target volume/clinical target volume.

^○^WHO grades I and II. Bold values provided are highlighting the overall results of patients numbers and outcome (Toxicity and tumor outcome).

## Re-Irradiation With Proton Therapy for Recurrent Meningioma After Radiotherapy

The Heidelberg group reported on 42 recurring patients who underwent prior RT for their meningioma (21). Compatible with the treatment policy of this center, most meningioma patients (34/42; 81%) were considered high-risk patients as a result of tumor volume, WHO grading or individual patient’s history, and were thus treated with carbon ion therapy. Only 8 (19%) patients were retreated with PT. For the entire cohort, the median follow-up time was 49.7, with a male/female gender ratio of 0.68. Most patients had a WHO grade II (*n* = 25) or III (*n* = 6) recurring tumor. Due to the limited number of patients, the analysis has been made with the combined carbon ion therapy and PT patients. The 1- and 2-year PFS were 71 and 56.5%, respectively. The median PFS for all patients was 34.3 months. Interestingly, the difference in PFS between WHO grades I and II–III tumors was significant (*p* = 0.03). The estimated median, 1- and 2-year OS was 61 months, 89.6 and 71.4%, respectively. Three (7.1%) patients presented with radiation-induced brain necrosis. All these patients presented with WHO grade II (*n* = 1) and III (*n* = 2) meningiomas and were retreated with 51 GyRBE delivered with carbon ions after an initial photon dose delivery of 54, 60 and 60 Gy, respectively. Additionally, four patients (9.5%) had worsening of their visual symptoms during follow-up.

Another series reported on the outcome of 16 recurring meningioma (WHO grade I, seven; grade II, eight and WHO grade 3, one) patients re-treated with PT (32). The median photon and proton dose for the initial treatment and for the re-irradiation was 54 Gy (range, 13–65.5) and 60 GyRBE (range, 30–66.6), respectively. After a median follow-up of 18.8 months after PT, 7 (44%) intracranial recurrences/progression were observed. The estimated 2-year RFS and OS were 43 and 94%, respectively. Patients with benign recurring meningioma had a significantly (*p* = 0.03) longer PFS than those with non-benign tumors. Of note, the late high-grade toxicity was substantial. Overall, 5 (31%) patients (median age, 72.9 years; range, 58.4–75.1) with WHO grade II (*n* = 4) and I (*n* = 1) tumors presented with late grade 3 toxicity, consisting of hydrocephalus (*n* = 3), seizures (*n* = 1) and carotid stenosis with consequentially cerebrovascular ischemia (*n* = 1). Three, one and one patients received 60, 59.4 and 54 GyRBE proton dose respectively for their re-treatment. No death resulting from this re-irradiation with protons was observed.

## Discussion

WHO grade I meningioma accounts for a substantial number of primary brain tumors in adults and are the most prevalent benign primary neoplasm of the brain. RT has been used with curative attempt in WHO grade meningioma patients whose tumors are not amenable to surgery, for subtotally resected (Simpson >III) tumors, for recurrent tumors or more rarely in the adjuvant setting (33). This treatment modality is an important component of the therapeutic armamentarium for meningioma delivered to mostly elderly patients. However, RT can result in a number of radiation-induced adverse events, including but not limited to cognitive impairment, pituitary dysfunction and secondary cancers. Regarding the former, a systematic review of 11 published series assessing the impact of surgery on the cognitive functioning of meningioma patients observed that most of these patients suffer from deficits in several cognitive domains comparative to the normative values (34). Interestingly, surgery seemed to improve cognitive function in most of these studies. Three series assessed the cognitive function of meningioma patients undergoing RT after surgery. Most of these studies (35, 36) but not all (37), showed that RT with or without surgery had an impact on the patients’ visual, verbal and working memory when compared to healthy controls, although the specific cognitive impairment attributable to RT alone was not assessed and no pre-treatment assessment of cognitive function was performed, which are both major disclaimers in the interpretation of these results. Nevertheless, it is safe to say that any radiation modality that could decrease the likelihood of cognitive impairment in elderly patients who are at risk of such a complication would be advisable.

The precise position of the Bragg peak (38) within the meningeal tumor has the possibility to decrease the dose delivered to critical structures within the brain and to decrease the overall brain integral dose. A recent dose comparative planning study assessing photon and proton therapy techniques for 20 meningioma >3 cm in size reported a mean dose and brain volumes receiving intermediate radiation dose (i.e. 20–30 Gy) approximately 50% lower (*p* ≤0.01) with intensity modulated PT (15). Additionally, the dose delivered to 40% of the bilateral hippocampus was significantly decreased by 74% in this study. These results are in line with other studies (39). Dose comparison analysis of PT *vs.* volumetric modulated arc photon radiotherapy have shown that integral doses were significantly (*p* <0.01) higher in all photon plans with a reduction of approximately 50% with PT (40). Substantial clinical evidence (41), while not unanimous (42), supports the notion that radiation-induced injury to the hippocampus may correlates with neurocognitive outcome of patients who are treated with RT. As such, PT may decrease the likelihood of long-term cognitive impairment and may be an option for elderly meningioma patients who are at greater risk of cognitive dysfunction or those younger patients with pre-existing clinically relevant neurocognitive impairment (**Table 3**).

**Table 3:** Indications for proton therapy in the management of WHO grades I–III meningioma.

Meningioma (WHO grade)	Treatment paradigm	Use of protons	Dose (GyRBE)	Level of evidence*	References
I (Benign)	Decrease in long term toxicity	Should be considered if clinically available for decreasing the probability of tumor induction	50.4–54	5	Bolsi et al. (43)
I (Benign)	Decrease in long term toxicity	Should be considered if clinically available for decreasing the probability of cognitive impairment	50.4–54	5	Florijn et al. (15)
II–III (Atypical/Malignant)	Dose escalation for tumor control	Should be considered if clinically available	>54.0	3b	McDonald et al. (30), Hug et al. (31), Boskos et al. (29)
Recurring (I–III)	Tumor control and mitigate the risk of radiation-induced adverse events	Should be considered if clinically available and especially if: * Non-elderly patient * Initial Benign histology * Previous irradiation at <60 Gy	≤60 (retreatment)	4	Imber et al. (32) El Shafie et al. (21)

^*^Oxford Centre for Evidence-Based Medicine. http://www.cebm.net/index.aspx?o=5653.

The survival of benign meningioma patients is substantial and secondary tumors may be observed after the delivery of adjuvant or radical radiation therapy (44). As such, any therapeutic modality that decreases the risk of radiation-induced tumors should be offered when appropriate. Chung et al. compared the reduction of secondary cancer risk in 558 pediatric and adult proton patients with matched photon patients identified in the SEER database (45). The observed secondary cancer incidence at 10 years was significantly decreased from 8.6% with photons to 5.4% with protons (Hazard ratio of 0.54; p <0.09). Importantly protons were delivered to patients with a passive scattering delivery paradigm that produced more neutrons than PBS (46, 47). The latter delivery may thus produce even less radiation-induced malignancies. It is noteworthy that the majority of meningioma patients managed with protons have been treated with a passive scattering delivery mode, as shown in **Tables 1** and **2**.

Radiation therapy is also a risk factor for developing radiation-induced meningioma and other tumors, and have been observed in 2.4–2.7% of patients in large case-series with a long (i.e. 20 years) follow-up period (48, 49). The use of conformal treatment such as PT may decrease the likelihood of developing secondary neoplasms by decreasing the low-bath dose delivered to the brain. The larger the meningioma is, the highest would be the theoretical advantage, as suggested by other authors (**Table 3**) (50, 51), for tumor induction and the aforementioned cognitive toxicity of this treatment modality. Theoretical tumor induction computations have shown in children that protons significantly decrease the risk of this unwanted complication (52), and the same effect has been suggested in adults with meningioma (43). Arvold et al. predicted a tumor-induction reduction of 50% with protons in a computational study on benign meningioma (39).

That being said, the potential benefit of PT has to be weighed against its substantial additional costs when compared to photon radiotherapy (53). Over 500 WHO grade I or image-defined meningioma have been treated with PT (**Table 1**). The clinical results (5-year LC >95% and toxicity rates) appear to be in line with the photon series. No cost effectiveness analysis has been made so far for PT delivered to this benign tumor. Based on the limited level of evidence (**Table 3**), PT can be considered for a benign meningioma patients if volumetrically challenging or if the patient has a higher risk of presenting radiation-induced toxicity after treatment.

WHO grade II and III meningiomas are tumors with poorer prognosis than benign meningiomas (54). Although the incidence has been substantially increased with the new 2016 WHO meningioma classification, it is still considerably lower than their benign counterparts. As such, the number of patients treated with protons for non-benign meningioma is substantially lower than for WHO grade I tumors by an order of 5 (**Tables 1**, **2**). These former tumors, especially WHO grade III meningiomas, show a local aggressive behavior, with or without distant brain or non-brain failures (55, 56). Although, the administration of radiation for WHO grade III tumors is certain, the role of radiotherapy for WHO grade II tumors is less clearly defined (57). Several survey have shown that only a minority of centers would recommend RT after Simpson I–III resection for WHO grade II meningioma (58). To address this important question, a phase III intergroup trial (ROAM; EORTC 1308) has been activated in 2016 randomizing Simpson 1–3 WHO grade II meningioma patients between observation and adjuvant RT delivering 59.4 Gy (59). This active study has an accrual target of 190 patients and over 60% of the patients have been currently accrued in this trial in Europe, Australia and New-Zealand. A systematic survey of 10 studies of adjuvant RT for grade II and III tumors showed that incomplete resection and dose delivered of <50 Gy were associated with a poorer 5-year PFS (60). Several retrospective analyses (**Table 2**) (29–31) of PT series have shown that increasing the delivered radiation dose may improve the patient outcomes. This parallels the experience with photon series (61–63). These dose–response observations in various analyses may validate the use of PT used with a dose-escalation paradigm (**Table 3**). A recent prospective European study (EORTC 22042-26042) has shown that the delivery of 60 Gy with photon-RT for Simpson I–III WHO grade II meningioma was associated with substantial toxicity, as grade 3–4 adverse event were observed in 10.7 and 3.6% of patients, respectively (64). This phase II-parallel non-randomized study assessed also the efficacy of high-dose radiotherapy in three other independent cohorts. Although the toxicity of the observational study for Simpson IV–V tumors treated with 70 Gy using photons has never been reported due to the small patients’ numbers, the toxicity of photon treatments at this dose level was notable (Weber DC, *personal communication*). As such, if moderate dose escalation is pursued, using the physical advantage of the proton beam’s properties to conform the dose deposition at a specific depth, the administration of PT in non-benign meningioma should be considered (**Table 3**). This has been the dose-strategy of all groups delivering PT for non-benign meningioma with doses up to 72 GyRBE (median/mean, 62–68) administered to these patients with no demonstrable increase the reported toxicity (**Table 2**). The reported outcome after PT is good, with a median 5-year LC of 52% for WHO grade II–III tumors (**Table 2**) but caution should be exercised not to over-estimate these results due to the small number (16–35; median 23) of patients and short follow-up intervals of those series (**Table 2**). The level of evidence justifying the administration of PT for non-benign meningioma, as with its benign counterpart, is low (**Table 3**).

The management of recurring or progressing meningioma after RT, especially high-grade tumors, is challenging. The therapeutic strategy is often limited but salvage options may include additional surgery and/or re-treatment with a radiation-modality such as brachytherapy (65, 66), photon radiotherapy including but not limited to normo- or hypo-fractionated radiotherapy/radiosurgery, and PT (32). Systemic therapy, including the administration of check-point inhibitors (67), is usually ineffective and rarely translates into radiological objective responses, although WHO grades II–III meningioma patients appear to benefit more from chemotherapy than whose with grade I disease (5, 68). Re-challenging these patients with radiation therapy again could potentially cause serious radiation-induced adverse events, as the organs at risk, including but not limited to the optic apparatus, brainstem and cochlea, have received a substantial dose of radiation already. As such, re-irradiation should be performed using highly conformal radiation techniques. The dose-deposition of particle therapy offers excellent sparing of organs at risk in direct vicinity of the recurrent tumor (**Figure 2**). Dose comparison analysis of PT *vs.* volumetric modulated arc photon radiotherapy have shown that integral doses were significantly (*p* <0.01) higher in all photon plans with a reduction of approximately 50% with PT (40) for recurring meningioma treated with re-irradiation. Using these techniques does not however nullify this risk, as illustrated in the Imber et al. study which reported a >30% rate of high-grade late radiation induced adverse events (32). Assessing the characteristics of these patients with late toxicity, it seems that age (median age, 72.9 years) and previous administered dose (median, 60 Gy) are important factors to consider when assessing the possibility of re-irradiating recurrent meningioma treated with prior RT with protons. Thus, one should consider PT for re-irradiation of non-elderly patients with recurring WHO grade I tumors treated previously with 50.4–54 Gy of radiation (**Table 3**), as those have the highest PFS and the lowest toxicity rates after re-irradiation. As for newly diagnosed or recurrent meningiomas treated with upfront PT, with or without surgery, the level of clinical evidence justifying the use of protons for re-irradiation is low (**Table 3**).

Regarding patient outcomes, most series reporting the results of PT for the management of WHO I and II–III tumors have shown that the local tumor control or survival of non-benign meningioma patients is lower than for patients with WHO grade I tumors (**Tables 1**, **2**). The Swiss group demonstrated that the 5-year LC rate was significantly lower (68 *vs.* 95.7%) for non-benign meningiomas when compared to WHO grade I tumors (22). This decrease in outcome-metrics is observed for non-benign meningiomas, even when these challenging tumors are treated with a dose-escalation paradigm (**Table 2**). This observed trend mirrors the results of modern photon RT series that report a PFS for atypical meningioma of approximately 70% (69). As such, we must be aware of developing a zealotry about PT for non-benign meningiomas and health providers must consider all existing published evidence before advocating protons for the management of these tumors. Possible explanation for this finding include but are not limited to the referral bias of large/recurrent tumors treated with PT, the span of multiple eras of proton technology (passive scattering, pencil beam scanning, intensity modulated PT, proton radiosurgery) and imbalances between the photon and proton groups with respect to known (age, gender, Simpson resection grade, tumor size, mitotic index) (69–71) and unknown baseline prognostic factors. Additionally, small patient numbers for this rare tumor and differences in patient cohorts between the photon and proton series complicate the interpretation of these findings.

PT is usually delivered to large and volumetrically complex meningiomas. The mean largest volume in the WHO grade 1 PT series is 193 cm^3^ (**Table 1**) and is usually larger than in photon series. It may be highly appropriate to treat these challenging patients with highly conformal radiation with a treatment modality that decreases the integral brain dose. The radiosurgery series have shown undisputedly that the largest the tumor volume is, the highest the likelihood of observing a radiation-induced adverse effect. A recent US series reporting on WHO grade I and II meningiomas treated with radiosurgery, has shown that patients who experienced cerebral edema were more significantly (p = 0–028) likely present with larger tumors on univariate analysis (72). These results are in line with other recent series (73–75). These clinical data legitimate thus the use of protons for selected meningioma patients with large tumors, especially if treated in a dose-escalation paradigm.

Visual toxicity and/or brain necrosis are classical complications of high-dose RT for the treatment of meningioma, too well known to merit a repeat citation here. It is reassuring to observe that proton series with a dose-escalation paradigm have not reported increased toxicity to the optic apparatus or brain (**Table 2**), when compared to photon series (76, 77). Taking the modern PT series, the observed rate of visual or brain toxicity is 0–1.5 and 2.9–4.5%, respectively (**Table 2**). In these PT series, radiation-induced toxicity was usually observed when the dose constraints were relaxed, such as those patients treated with 63.2–67.4 GyRBE to the optic apparatus (26). It is important however to note that adverse events occur with photon or proton radiation even when the dose constraints are consciously met and when the patient has no risk factors (76). In the Swiss series, the only grade 5 brain necrosis occurred in a WHO grade I patient treated with 54 GyRBE with all dose-constraints met. In the same series, the two optic nerve and the two other retinopathy observed were also planned respecting all optic apparatus dose constraints. Regarding the toxicity on these organs at risk, several groups have tried to identify dose metrics predictive of clinically relevant toxicity (78–82) but the results are not robust and the implementation of these constraints is problematic in some patients with WHO grade II or III tumors. Care should be taken that patients should be aware of these rare adverse events in the informed consent process that should adhere to national guidelines.

Our review has a number of limitations that future studies should address. Firstly, all reviewed data are retrospective in nature and are thus subject to a known and unknown biases that have been only partially discussed in this section of the manuscript. The reviewed data lacked prospectively captured patient-reported outcomes and quality of life that are important in the area of modern neuro-oncology. PSI will submit soon the analysis of the QoL of meningioma patients treated with protons using the EORTC C30 and BN20 questionnaires. These data will also enhance substantially our understanding of how exactly PT may distinguish itself clinically from other radiation modalities. Prospective cohort studies from other institutions will greatly benefit our understanding of meningioma patient outcomes treated with protons and are arguably required at this juncture in time. The concept of an international prospective registry, such as the one proposed by the European Particle Therapy Network in its Clinical Work-package 1 (83), performed under a standardized protocol is immeasurably desirable, for it will allow more homogenous data to accumulate from multiple European experiences. These could then bolster our ability to perform more robust bias assessments, assess the true value of protons for this indication and justify any decision-algorithms, with or without cost–benefit analyses. Finally, the relatively short follow-up time and more importantly the limited number of patients in the cohorts limit somehow the generated level of evidence, especially so for secondary malignancies.

## Conclusions

The delivery of PT for the treatment of intracranial meningioma may be discussed in clinical settings including but not limited to volumetrically challenging tumors, non-benign histology or for the re-irradiation of recurring/progressive tumors. Patient with a high-risk of radiation induced toxicity may also benefit from the decrease of dose delivered to critical structures such as the optic apparatus and the brain. The outcome of approximately 500 WHO grade I meningioma patients have been reported with excellent tumor control rates and rare radiation-induced adverse events. For WHO grade II–III meningiomas treated with a dose-escalation paradigm, the toxicity profile is clinically acceptable. Re-irradiation of progressing/recurring tumors with protons should be discussed on a case to case basis and should be limited to those younger patients with benign tumors that should most benefit from protons.

## Author Contributions

DW, AB, and MJ wrote the paper. NB provided the figures and Legends and wrote one section of the MS. All authors contributed to the article and approved the submitted version.

## Conflict of Interest

The authors declare that the research was conducted in the absence of any commercial or financial relationships that could be construed as a potential conflict of interest.
